# Draft Genome Sequence of Alkaliphilic Exiguobacterium sp. Strain HUD, Isolated from a Polymicrobial Consortia

**DOI:** 10.1128/genomeA.01451-14

**Published:** 2015-01-22

**Authors:** Simon P. Rout, Anup Rai, Paul N. Humphreys

**Affiliations:** Department of Biological Sciences, School of Applied Sciences, University of Huddersfield, Queens Gate, Huddersfield, United Kingdom

## Abstract

An alkaliphilic microorganism from the genus Exiguobacterium, Exiguobacterium sp. strain HUD was isolated from a fermentative, methanogenic polymicrobial microcosm operating at pH 10. The draft genome shows the presence of genes encoding for the metabolism of a range of carbohydrates under both aerobic and anaerobic conditions.

## GENOME ANNOUNCEMENT

Members of the genus Exiguobacterium, non-spore-forming Gram-positive microorganisms within the phylum *Firmicutes* were first described by Collins et al in 1983 (1). Exiguobacterium spp. have been isolated from a range of habitats including: permafrost, glaciers, soils, fresh and salt waters, hydrothermal vents, and brine shrimp (2). Their presence in these diverse environments is reflected in their ability to survive and grow in extremes of temperature (−12 to 55°C) and pH (5 to 11) and to survive under stresses generated by UV irradiation (3), antibiotics (4), and heavy metals (5, 6). Members of this genus are also noted for their ability to utilize a range of substrates, particularly for the bioremediation of azo dyes, and as a result of this, the reservoir of enzymes produced by these organisms has received considerable attention (7, 8).

Here, we present the draft genome sequence of Exiguobacterium sp. HUD isolated from an anaerobic microcosm operating at pH 10 described previously (S. P. Rout, C. J. Charles, C. Doulgeris, A. J. McCarthy, D. J. Rooks, P. Loughnane, A. P. Laws, and P. N. Humphreys, unpublished data), where products from the anaerobic alkaline degradation of cellulose, including the α and β forms of isosaccharinic acid, were used as a carbon source. The original inoculum for the microcosm was obtained from a mesophilic near surface anaerobic sediment from a canal (Huddersfield, United Kingdom). Microcosm effluent was used as an inoculum, where 10 µL of suspension was streaked out onto fastidious anaerobic agar (pH 10, LabM, United Kingdom) under a stream of nitrogen and incubated under anaerobic conditions (10% H_2_:10% CO_2_: 80% N_2_; DW Scientific, United Kingdom) for 48 h. A single colony was selected from the plate and purified through further subculture before total genomic DNA was isolated using a commercial kit (Ultraclean microbial isolation kit; Mo-Bio, USA).

A draft whole-genome sequence was obtained using a whole-genome shotgun (WGS) sequence strategy. Paired-end 125 cycles sequence reads were generated using the Illumina HiSeq 2500 system (BaseClear, The Netherlands). FASTQ sequence files were generated using the Illumina Casava pipeline version 1.8.3 and the assembly prepared using CLC Genomics Workbench version 7.0.4. The contigs were linked and placed into scaffolds or supercontigs. The orientation, order, and distance between the contigs was estimated using the insert size between the paired-end and/or mate-pair reads using the SSPACE Premium scaffolder version 2.3 (9). Whole-genome sequencing generated 826 contigs with a draft genome 3,359,295-bp in length and G+C content of 51.1%. The draft genome contained a total of 3,484 coding sequences (CDS), where 19 pseudogenes, 9 genes coding for rRNA (5S, 16S, 23S), 69 genes coding for tRNAs, and 1 noncoding RNA (ncRNA) were present. Further analysis using RAST (10) revealed the presence of a number of genes encoding proteins involved in both aerobic and anaerobic carbohydrate metabolism. As previous authors have noted with other members of this genus, genes encoding stress response proteins were also observed (11). The genome also suggests resistance to a range of metals (As, Cd, Cr, Hg) as well as the potential for multidrug resistance.

### Nucleotide sequence accession number.

This whole-genome shotgun project has been deposited at DDBJ/EMBL/GenBank under the accession no. JQGI00000000.

## References

[1] Collins MD, Lund BM, Farrow JAE, Schleifer KH 1983 Chemotaxonomic study of an alkalophilic bacterium, *Exiguobacterium aurantiacum* gen. nov., sp. nov. Microbiology 129:2037–2042. doi:10.1099/00221287-129-7-2037.

[2] Vishnivetskaya TA, Kathariou S, Tiedje JM 2009 The *Exiguobacterium* genus: biodiversity and biogeography. Extremophiles 13:541–555. doi:10.1007/s00792-009-0243-5. 19381755

[3] Ordoñez OF, Flores MR, Dib JR, Paz A, Farías ME 2009 Extremophile culture collection from Andean lakes: extreme pristine environments that host a wide diversity of microorganisms with tolerance to UV radiation. Microb Ecol 58:461–473. doi:10.1007/s00248-009-9527-7. 19495855

[4] Yang J, Wang C, Wu J, Liu L, Zhang G, Feng J 2014 Characterization of a multiresistant mosaic plasmid from a fish farm sediment *Exiguobacterium* sp. isolate reveals aggregation of functional clinic-associated antibiotic resistance genes. Appl Environ Microbiol 80:1482–1488. doi:10.1128/AEM.03257-13. 24362420 PMC3911065

[5] Alam MZ, Malik A 2008 Chromate resistance, transport and bioreduction by *Exiguobacterium* sp. ZM‐2 isolated from agricultural soil irrigated with tannery effluent. J Basic Bicrobiol 48:416–420. doi:10.1002/jobm.200800046. 18759228

[6] Belfiore C, Ordoñez OF, Farías ME 2013 Proteomic approach of adaptive response to arsenic stress in *Exiguobacterium* sp. S17, an extremophile strain isolated from a high-altitude Andean lake stromatolite. Extremophiles 17:421–431. doi:10.1007/s00792-013-0523-y. 23525943

[7] Dhanve RS, Shedbalkar UU, Jadhav JP 2008 Biodegradation of diazo reactive dye Navy blue HE2R (reactive blue 172) by an isolated *Exiguobacterium* sp. RD3. Biotechnol Bioprocess Eng 13:53–60. doi:10.1007/s12257-007-0165-y.

[8] Arora PK, Sharma A, Mehta R, Shenoy BD, Srivastava A, Singh VP 2012 Metabolism of 4-chloro-2-nitrophenol in a Gram-positive bacterium, *Exiguobacterium* sp. PMA. Microb Cell Fact 11:150. doi:10.1186/1475-2859-11-150. 23171039 PMC3539986

[9] Boetzer M, Henkel CV, Jansen HJ, Butler D, Pirovano W 2011 Scaffolding pre-assembled contigs using SSPACE. BioInformatics 27:578–579. doi:10.1093/bioinformatics/btq683. 21149342

[10] Aziz RK, Bartels D, Best AA, DeJongh M, Disz T, Edwards RA, Formsma K, Gerdes S, Glass EM, Kubal M, Meyer F, Olsen GJ, Olson R, Osterman AL, Overbeek RA, McNeil LK, Paarmann D, Paczian T, Parrello B, Pusch GD 2008 The RAST server: Rapid Annotations using Subsystems Technology. BMC Genomics 9:75. doi:10.1186/1471-2164-9-75. 18261238 PMC2265698

[11] Ponder MA, Gilmour SJ, Bergholz PW, Mindock CA, Hollingsworth R, Thomashow MF, Tiedje JM 2005 Characterization of potential stress responses in ancient Siberian permafrost psychroactive bacteria. FEMS Microbiol Ecol 53:103–115. doi:10.1016/j.femsec.2004.12.003. 16329933

